# Dominantly Inherited Hereditary Nonpolyposis Colorectal Cancer Not Caused by MMR Genes

**DOI:** 10.3390/jcm9061954

**Published:** 2020-06-23

**Authors:** Mariona Terradas, Gabriel Capellá, Laura Valle

**Affiliations:** 1 Hereditary Cancer Program, Catalan Institute of Oncology, IDIBELL, Hospitalet de Llobregat, 08908 Barcelona, Spain; mterradas@idibell.cat (M.T.); gcapella@idibell.cat (G.C.); 2 Program in Molecular Mechanisms and Experimental Therapy in Oncology (Oncobell), IDIBELL, Hospitalet de Llobregat, 08908 Barcelona, Spain; 3 Centro de Investigación Biomédica en Red de Cáncer (CIBERONC), 28029 Madrid, Spain

**Keywords:** hereditary cancer, colorectal cancer, mismatch repair proficiency, familial colorectal cancer type X, gene identification, cancer predisposition, cancer susceptibility, cancer genetics, molecular pathways

## Abstract

In the past two decades, multiple studies have been undertaken to elucidate the genetic cause of the predisposition to mismatch repair (MMR)-proficient nonpolyposis colorectal cancer (CRC). Here, we present the proposed candidate genes according to their involvement in specific pathways considered relevant in hereditary CRC and/or colorectal carcinogenesis. To date, only pathogenic variants in *RPS20* may be convincedly linked to hereditary CRC. Nevertheless, accumulated evidence supports the involvement in the CRC predisposition of other genes, including *MRE11*, *BARD1*, *POT1*, *BUB1B*, *POLE2*, *BRF1*, *IL12RB1*, *PTPN12*, or the epigenetic alteration of *PTPRJ*. The contribution of the identified candidate genes to familial/early onset MMR-proficient nonpolyposis CRC, if any, is extremely small, suggesting that other factors, such as the accumulation of low risk CRC alleles, shared environmental exposures, and/or gene–environmental interactions, may explain the missing heritability in CRC.

## 1. Introduction

**Figure 1:**
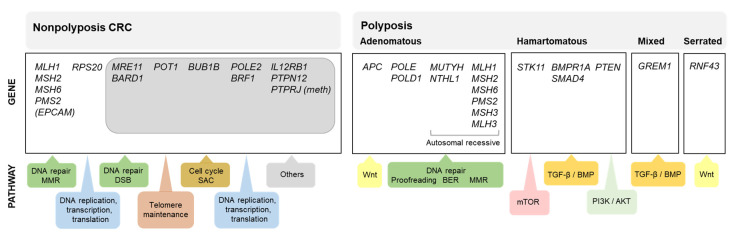
Schematic representation of colorectal cancer predisposing syndromes, causal genes, and affected molecular pathways. Genes shaded in grey correspond to the most promising proposed candidate genes. Abbreviations: BER, base excision repair; DSB, double strand breaks; meth, promoter hypermethylation; MMR, DNA mismatch repair; SAC, spindle assembly checkpoint.

While estimates indicate that approximately 14% of all colorectal cancer (CRC) patients have at least one first-degree relative affected with the same tumor type [1,2], 4–8% of all CRC patients carry germline pathogenic variants in one of the known high penetrance genes for this tumor [3,4,5,6], with a relevant proportion of the familial aggregation of CRC remaining unexplained. The identification of a germline pathogenic variant in a colorectal cancer-predisposing gene has important consequences for the patients and their relatives, as they can be counseled and managed based on gene-specific guidelines. This is the main reason why large efforts have been and are still being made to identify the genetic cause of the increased CRC risk observed in some families. Figure 1 shows the known causal genes for nonpolyposis and polyposis CRC predisposition and the molecular pathways in which they are involved.

Classically, hereditary cancer studies were mainly based on genome-wide linkage analysis of large individual or multiple pedigrees followed by positional cloning and the study of somatic studies. This strategy led to the identification of the most prominent hereditary cancer genes, including the main genes involved in nonpolyposis colorectal cancer predisposition, i.e., the DNA mismatch repair (MMR) genes *MLH1*, *MSH2*, *MSH6,* and *PMS2*; however, it seemed unable to identify additional causal genes for hereditary nonpolyposis CRC (HNPCC). In fact, while genome-wide linkage studies in families with CRC allowed the identification of several dominant predisposition loci mapped to different chromosomal regions, no evident causal genes have been identified within these loci [7]. The poor performance of these techniques for the identification of additional high-penetrant genes responsible for familial CRC cases could have been caused by the heterogeneity of the clinical group, the existence of oligo- or polygenic modes of inheritance, or the presence of unconventional mechanisms of gene inactivation, among other possibilities.

Thanks to the rapid development of massively parallel sequencing-based approaches and genome-wide copy number techniques, genome sequencing, exome sequencing, or genome-wide scanning of copy number alterations, alone or in combination with linkage analysis, and applied to isolated high-risk families or to multiple families or probands with common phenotypes, are being used for the identification of causal pathogenic variants. This type of study, performed in the last decade, has resulted in the identification of numerous candidate causal genes for the nonpolyposis CRC predisposition. In parallel to these a priori agnostic analyses, candidate gene studies have been performed along the years to assess the involvement of specific genes selected based on their function or involvement in molecular pathways deemed relevant in colorectal carcinogenesis.

Here, we aimed to provide an overview of the proposed candidate causal genes for hereditary colorectal cancer based on different molecular entities and focused on relevant molecular pathways.

## 2. *RPS20* Mutations as a Rare Cause of Hereditary Nonpolyposis Colorectal Cancer

So far, the only new candidate gene that has shown consistent association with hereditary nonpolyposis CRC is *RPS20*, which encodes a component (S20) of the small ribosome subunit. By combining genetic linkage analysis and exome sequencing, Nieminen et al. (2014) identified a novel truncating *RPS20* variant, c.147dupA (p.Val50Serfs*23), in a Finnish four-generation CRC-affected family. The variant was present in seven CRC-affected members but neither in four cancer-free members nor in one relative diagnosed with breast cancer at age 55. All studied tumors were MMR proficient and despite not showing loss of the *RPS20* wildtype allele, patients carrying the *RPS20* c.147dupA variant showed a marked increase of 21S pre-rRNAs, supporting a late pre-rRNA processing defect consistent with haploinsufficiency. No additional *RPS20* (likely) pathogenic variants were identified in 25 Finnish MMR-proficient Amsterdam-positive families and in 61 primary CRCs and cancer cell lines [8].

Broderick et al. (2017) analyzed by exome sequencing 863 early onset/familial CRC cases and 1604 individuals without CRC and no germline mutations in known hereditary CRC genes. The authors identified a truncating *RPS20* variant, p.Leu61Glufs*11, in a 39-year-old individual with metachronous CRC. They also identified a predicted pathogenic missense variant, p.Val54Leu, in a CRC patient diagnosed with CRC at age 41, who fulfilled the Amsterdam criteria for hereditary CRC. No rare missense or disruptive *RPS20* variants were detected in the 1604 controls [9]. Very recently, we performed a mutational screening of *RPS20* in 473 familial/early onset CRC cases and did not identify any predicted pathogenic variant. Taking the three studies together, we concluded that disruptive (stop-gain, frameshift, and start-loss) variants are enriched in familial/early onset CRC cases compared to controls [10]. Supporting this association with hereditary CRC, *RPS20* c.177+1G>A has recently been identified in another family with four CRC-affected members, all of them carriers or obligate carriers of the *RPS20* variant [11]. The limited available data suggests low prevalence (allele frequency in familial/early onset CRC patients: 2/2,724; 0.07%) and high penetrance (13/16 (81%) > 35-year-old carriers of disruptive or canonical splice-site variants were affected with CRC) for *RSP20* pathogenic variants, as well as the absence of extracolonic manifestations. Data from additional carriers are required to estimate risks and recommend gene-specific surveillance measures. 

## 3. Candidate Causal Genes for Mismatch Repair Proficient Hereditary Nonpolyposis Colorectal Cancer

In this section, we introduce the candidate genes proposed these past years for CRC predisposition. To date, the evidence gathered is not enough to include any of these genes in routine genetic testing. Additional studies will provide additional insight about their causal association, and if confirmed, they will provide information about the associated cancer risks. In the following subsections, the genes are presented according to gene ontology (molecular pathway). We tried to include the most relevant studies for each gene; however, we would like to apologize in advance to those whose work has not been cited due to space constraints. Table S1 gathers information on the proposed candidate genes together with the related literature.

### 3.1. DNA Damage Response

Defects in DNA repair mechanisms are directly associated with cancer development. Germline pathogenic variants in genes coding for DNA mismatch repair (MMR) proteins cause Lynch syndrome, the most prevalent form of hereditary nonpolyposis CRC. Germline alterations in other genes involved in the DNA damage response (DDR) have been proposed as genes potentially involved in CRC predisposition [5,12,13,14]. DDR is a complex defense system whose aim is to detect, signal, and promote the repair of DNA lesions. DDR mechanisms include the regulation of the cell cycle, transcription programs and chromatin accessibility, and, when DNA damage is massive, the activation of cell fate pathways, such as apoptosis or senescence [15,16,17,18]. In the next subsections, we will introduce the genes involved in DDR that have been proposed as candidate causal genes for CRC predisposition.

#### 3.1.1. DNA Repair

##### Base Excision Repair

The base excision repair (BER) mechanism corrects oxidative DNA damage, one of the hallmarks of cancer [19]. Several genes involved in BER, such as *MUTYH* and *NTHL1*, when mutated in the germline in a dominant recessive manner, cause CRC and adenomatous polyposis predisposition [20,21]. Based on this, other BER genes have been considered good candidates for CRC predisposition.

For years, several groups undertook the study of *OGG1* and *NUDT1* (= *MTH1*) variants as potential causal factors of CRC predisposition. Several studies have been published, and what seemed to be promising mostly in the first years [22,23,24,25,26], reduced their degree of interest, at least as high- or moderate-risk genes, when subsequent larger studies suggested no causal association [27,28,29].

Other BER genes, such as *NEIL2*, *TDG*, and *UNG*, among others, have also been studied as potential genes for CRC predisposition; however, the evidence gathered suggest that their role is negligible [28,30,31] (Table S2).

##### Nucleotide Excision Repair and *MGMT*

Nucleotide excision repair (NER) is mainly involved in the removal of bulky adducts that results from UV DNA damage. While biallelic mutations in the main NER genes are linked to xeroderma pigmentosum, heterozygous likely pathogenic missense variants in *XPC*, *ERCC2*, and *ERCC6* have been identified in CRC patients [12,32,33]. Additional studies will determine whether the identified variants are or are not causally associated with an increased CRC risk.

MGMT (O6-methylguanine DNA methyltransferase) is a DNA repair enzyme in charge of removing potentially mutagenic alkyl groups primarily from the O6-position of guanine molecules. MGMT activity is essential for genome integrity given that it prevents mismatch, replication, and transcription errors, which may lead to carcinogenic and apoptotic events. Based on MGMT’s role in DNA repair and the fact that *MGMT* epigenetic silencing has been reported as an early event in CRC [34,35], our group decided to test its involvement in CRC predisposition. While no constitutional epimutations were identified, 4 rare heterozygous missense variants were identified in 6 of the 473 familial/early onset unrelated CRC patients studied. Two variants were clearly predicted as benign and the other two, p.His116Tyr and p.Arg159Gln, were further studied. None of the two caused a statistically significant reduction of MGMT repair activity and no evidence of somatic second hits was found in the studied tumors. Case-control data showed over-representation of c.346C > T (p.His116Tyr) in familial CRC compared to controls, but no overall association of *MGMT* mutations with CRC predisposition [36].

##### Double-Strand Break Repair

Double-strand breaks (DSBs) are considered the most deleterious form of DNA damage. DSB are the base of break-fusion-bridge cycles; i.e., the engine of chromosome instability, which is a form of genomic instability that characterizes MMR-proficient CRC tumors [37,38,39]. Therefore, it is not surprising that germline (likely) pathogenic variants in genes involved in DSB repair—in particular, in homologous recombination (HR) and non-homologous end joining (NHEJ)—had been identified in CRC patients. 

*MRE11* encodes an endonuclease member of the MRN complex (MRE11, Rad50, and NBS1) in charge of sensing and promoting DSB end resection during HR. Chubb et al. (2016) identified six carriers of rare or novel variants in *MRE11,* either disruptive or predicted pathogenic missense variants, among 1006 familial/early onset CRC patients, compared to one predicted pathogenic variant identified in 1609 controls [28]. Aldubayan et al. (2018) identified two additional carriers of *MRE11* predicted pathogenic missense variants among 667 CRC patients [5], and Belhadj et al. (2020) 2 more carriers in a 473-familial/early onset-CRC cohort. A meta-analysis of all reported series compared to a control population indicated that *MRE11*-disruptive variants are significantly enriched in familial/early onset CRC, supporting the role of *MRE11* in CRC predisposition [10]. On the other hand, it has been suggested that *MRE11* and other MRN components may be used as biomarkers for predicting disease progression and treatment response. In particular, low MRE11 expression has been associated with improved oxaliplatin sensitivity and better progression-free survival in CRC patients [40,41], which might be translated to the treatment and clinical impact of the tumors developed in the context of a germline *MRE11* pathogenic variant.

Díaz-Gay et al. (2019) recently carried out an integrated analysis of germline and tumor exome sequencing data in 18 high-risk CRC families, with the aim of identifying new candidate genes for hereditary colorectal cancer. The authors followed a prioritization strategy based on the selection of genes affected by two hits, one germline and one somatic, according to Knudson’s hypothesis, i.e., genes susceptible to having a tumor suppression growth effect. In total, 7 out of the 16 identified candidates belonged to DNA repair pathways, and 4 of them were involved in DSB repair: *BRCA2*, *RIF1*, *BLM*, and *RECQL*. *BRCA2* is a classical non-CRC cancer predisposition gene that predisposes to hereditary breast and ovarian cancer and its implication in HNPCC will be discussed in Section 4. *RIF1* encodes a protein that localizes to aberrant telomeres and is recruited to DSBs to counteract DNA resection, thus promoting repair by NHEJ. The authors identified a predicted pathogenic *RIF1* missense variant and tumor LOH in the proband of one of the studied CRC families [33]. As no additional evidence has been reported, we looked up *RIF1* in the exome sequencing data reported by Chubb et al. (2016). No disruptive and 19 predicted pathogenic missense variants were identified in 1006 familial/early onset CRC patients (1.9%) compared to 39 predicted pathogenic missense variants identified in 1609 healthy controls (2.4%), suggesting no association with cancer predisposition [28].

*BLM* encodes a RecQ-like helicase that participates in the final stages of HR. While biallelic pathogenic variants cause Bloom syndrome (Mendelian Inheritance in Man (MIM)# 210900), the question of whether monoallelic pathogenic variants predispose to CRC has been debated for almost two decades. Gruber et al. (2002) first noticed that heterozygous carriers of a recurrent *BLM* Ashkenazi pathogenic variant (*BLM*^Ash^) were at increased risk of developing CRC [42]. Since then, numerous studies have been published, some of them supporting the association of heterozygous *BLM* pathogenic variants with various types of cancers, including CRC [43,44,45,46,47,48,49,50], and others where no association with increased cancer risk was detected [5,51,52,53,54]. A recent meta-analysis combining our own *BLM* mutational screening with previous studies and publicly available sequencing data from familial and/or early onset CRC patients suggested a lack of association of *BLM* heterozygous disruptive and predicted pathogenic variants with CRC predisposition after comparison with the frequencies in population controls [10].

In addition to *BLM*, other RecQ helicases have been linked to CRC predisposition. Homozygous or compound heterozygous mutations in *WRN* cause Werner syndrome (MIM# 277700), a rare segmental progeroid syndrome characterized by chromosomal instability and cancer predisposition. Moreover, somatic *WRN* mutations are identified in 4% of colorectal tumors and in other cancers [55]. Rare or novel heterozygous germline variants were identified in MMR-proficient familial/early onset CRC patients [12,55]; however, no enrichment of *WRN* disruptive and/or predicted pathogenic variants are detected in cases (36/1006; 3.6%) compared to controls (82/1609; 5.1%) [28], suggesting no association with the disease. On the other hand, monoallelic frameshift mutations in *RECQL4*, a gene that causes the autosomal recessive Rothmund Thomson syndrome (MIM# 268400), have been found in 2 of 680 unselected CRC patients [5]. The exome/genome sequencing results from Chubb et al. showed that no enrichment of disruptive *RECQL4* variants is found in cases (3/1006) compared to controls (5/1609), with similar results when including predicted pathogenic variants (Table S2) [28]. In summary, despite the identification of rare or novel heterozygous variants among CRC patients, current evidence does not support a causal role of RecQ helicases in CRC predisposition, at least not as high penetrance genes.

Variants in other genes involved in DSB repair have been identified in the germline in CRC patients. Disruptive and canonical splice-site germline variants have been detected in *BARD1*, a gene that encodes an HR-related protein [5,13]; however, its involvement as a high penetrance gene remains controversial [56]. These types of variants in *BARD1* occur more frequently in familial/early onset CRC patients (5/1006; 0.5%) than in controls (2/1609; 0.12%) [28]; however, additional evidence is needed to elucidate the role of *BARD1* in hereditary CRC. Homozygous variants in *MCM9*, a DNA helicase involved in HR, DNA replication and MMR, were described in two polyposis-affected siblings [57]. However, recent case-control data suggests a lack of association of homozygous or heterozygous variants with polyposis or CRC predisposition [10,58]. A frameshift mutation in *XRCC4*, a member of the DNA ligase 4 complex involved in the last step of NHEJ, was found in a CRC patient with familial CRC history, which, together with the lack of *XRCC4* variant carriers among population controls, led the authors to propose *XRCC4* as a candidate gene for CRC predisposition [13]. Nonetheless, sequencing data from cases and controls does not show over-representation of predicted pathogenic variants in familial/early onset CRC patients (8/1006) compared to controls (11/1609) [28]. Predicted pathogenic variants in *POLQ*, a DNA polymerase involved in the alternative DSB repair pathway θ-mediated end joining (TMEJ), were found in familial CRC and polyposis patients [48,59], but studies in additional cohorts and combined analysis of available data did not support this association [10,60].

##### Fanconi Anemia Pathway

Due to their involvement in DNA repair, several groups have studied or focused their genome-wide results on variants affecting genes involved in the Fanconi anemia pathway, such as *BRCA2/FANCD1*, *BRIP1/FANCJ*, *FANCC*, *FANCE*, and *REV3L/POLZ* [9,60,61]. Functional and co-segregation studies identified *FAN1* as a promising CRC predisposing gene; however, more recent case-control studies have found no enrichment of disruptive, canonical-splicing, and predicted pathogenic missense variants in CRC cases compared to controls [9,10]. Nevertheless, available evidence suggests that *FAN1* c.149T > G (p.Met50Arg) might increase the risk to CRC and possibly to other tumor types [10].

#### 3.1.2. Telomere Maintenance

Maintenance of telomeres is essential to chromosome stability. Unprotected telomeres are recognized by the DNA repair machinery as DSBs, and illegitimate repair between chromosome ends or with an unrepaired DSB results in chromosome reorganizations. *POT1* codes for one of the components of the telomere shelterin complex, having a critical function in genome stability. In fact, this gene shows a significant intolerance to loss-of-function variants (GnomAD v.2.1.1: LOEUF = 0.362). In the past years, multiple reports associated *POT1* germline variants with a predisposition to various types of tumors, including CRC [10,28,62,63,64,65,66]. Chubb et al. identified two carriers of disruptive predicted pathogenic *POT1* variants in 1006 CRC patients, while no disruptive variants were identified in 1609 controls [28]. We identified, among 473 familial/early onset CRC patients [10], a predicted pathogenic missense variant that had been previously associated with an increased risk of chronic lymphocytic leukemia [67]. Taking all available studies into account, we may now preliminary conclude that CRC is part of the tumor spectrum of the *POT1* cancer-predisposing syndrome.

#### 3.1.3. Cell Cycle—Checkpoint and Chromosome-Associated Proteins

Cell cycle dysregulation may drive tumorigenesis; therefore, it is not surprising that germline variants in genes coding for cell cycle checkpoint proteins, including factors involved in the proper formation and segregation of chromosomes, have been identified in CRC patients. 

Gene variants in several components of the spindle assembly checkpoint (SAC), which ensures proper chromosome segregation during mitosis, have been associated with CRC predisposition, including *BUB1B*, *BUB1*, *BUB3*, and *CDC27* [68,69,70,71]. Biallelic mutations in the SAC component *BUB1B* had been classically linked to mosaic variegated aneuploidy (MVA). In 2010, Rio Frio et al. reported a patient with MVA who had developed an ampulla of Vater at 34 years of age and two decades later, adenomatous polyps at the gastrointestinal tract and multiple primary invasive adenocarcinomas of the colon and the stomach. He carried a homozygous intronic mutation, c.2386-11A > G, that creates a de novo splice site resulting in low levels of BUBR1 protein (encoded by *BUB1B*) [70]. However, no biallelic *BUB1B* rare variants were subsequently identified among 192 individuals with early onset CRC, indicating that the biallelic *BUB1B* pathogenic hardly ever occurs in the germline of individuals with CRC [72]. Moreover, sequencing data from Chubb et al. identified only one carrier of a heterozygous disruptive *BUB1B* variant (and no predicted pathogenic missense variants) among 1006 familial/early onset CRC patients and none among controls [28], further supporting the rarity of these alterations.

Using genome-wide copy number profiling and exome sequencing in early onset and familial CRC, De Voer et al. identified six germline alterations in *BUB1* and *BUB3* affecting six independent CRC families. Carriers had variegated aneuploidies in multiple tissues and variable dysmorphic features [68]. Broderick et al. found no increased frequency of *BUB1* and *BUB3* mutations in cases compared to controls [9]. Recently, Mur et al. identified three *BUB1* and one *BUB3* rare germline variants among 456 MMR-proficient familial/early onset CRC and 88 polyposis patients. Neither variegated aneuploidy nor dysmorphic traits were observed in carriers; however, one of the variants showed evident in vitro functional effects [69].

DeRycke et al. [71] performed exome sequencing in 16 families affected with CRC, identifying several predicted pathogenic variants in genes coding for mitotic factors, such as *CDC27*, *DDX12*, *HAUS6/FAM29A*, *HIST1H2BE*, *TACC2*, and *ZC3HC1*, and paying special attention to *KIF23* and *CENPE*, located within previously reported CRC linkage regions [73]. Tanskanen et al. performed exome sequencing in 22 early onset CRC patients, using additional exome sequence data from 95 familial CRC patients as a validation set. They identified two frameshift variants in *SYNE1* and homozygous variants in *DONSON*; both genes being related to the cell cycle [74]. Despite the interest of these genes, no further studies have been performed to decipher their causal role in CRC predisposition. None of the genes mentioned in this paragraph, except *TACC2* (3/1006 disruptive variants in CRC patients compared to 1/1609 in controls), showed an enrichment of germline disruptive and/or predicted pathogenic variants in cases compared to controls (Table S2) [28].

### 3.2. DNA Replication, Transcription, and Translation

In 2015, Spier et al. described for the first time the presence of germline variants in *POLE2*, a member of the DNA polymerase epsilon complex, as a potential cause of CRC predisposition. Specifically, they found a predicted pathogenic missense variant in a polyposis patient and a stop-gain variant in an individual with an unknown phenotype [75]. This stop-gain variant was also observed by Chubb et al. in 5 of 1006 familial/early onset CRC and absent in 1609 controls, together with two missense predicted pathogenic variants in three additional CRC patients [28]. In light of these promising findings, we performed a mutational screening of *POLE2* in 473 familial/early onset CRC cases, finding 4 additional carriers of predicted pathogenic missense variants. In the same study, a meta-analysis considering all available data showed that disruptive and canonical splice-site variants in *POLE2* are over-represented in familial/early onset CRC cases compared to controls [10]. It has recently been reported that depletion of B-family DNA polymerases, which includes *POLE2*, together with CHK1 pharmacological inhibition is a synthetically lethal combination in human colorectal cancer cells, which opens a promising window of opportunity for the treatment of *POLE2*-derived tumors [76].

Our group identified a germline splicing variant (c.1459+2T > C) in *BRF1*, which encodes an RNA polymerase III transcription initiator factor subunit, in three CRC-affected members of an Amsterdam I family. Mutational screening of *BRF1* in 503 CRC families identified a total of 11 novel or rare germline variants; a significant larger proportion than in the control population. Seven of the identified variants affected *BRF1* mRNA splicing, protein stability, or expression and/or function [56]. Exome sequencing data from Chubb et al. shows that, although statistically non-significant and infrequent, predicted pathogenic variants are more frequent among familial/early onset CRC patients (2/1006; 0.2%) that in controls (2/1609; 0.12%) [28].

Finally, rare germline variants in transcription- or translation-associated genes have also been identified in CRC patients, including variants in *CTBP2*, *IRF5*, *MED12*, *RNF111*, *SF1*, *TLE1*, *TLE4*, and *TRIP4* [71] or *ZNF490* and *MRPL3* [77]. Exome sequencing data in 1006 early onset CRC patients and 1609 controls indicates that no enrichment of disruptive or predicted pathogenic variants in any of the mentioned genes is found in cases compared to controls (Table S2) [28].

### 3.3. Wnt and TGF-beta Pathways

Most of the known hereditary colorectal cancer genes that are not involved in DNA repair, causing either polyposis or nonpolyposis phenotypes, affect three very specific signaling pathways: Wnt (*APC, RNF43, AXIN2*), TGF-beta/BMP (*SMAD4, BMPR1A, GREM1*), or PI3K/AKT/mTOR (*STK11, PTEN*) (reviewed in [78]). Therefore, other genes involved in those pathways have been considered good candidates for CRC predisposition.

*LRP6* encodes a component of the Wnt-Fzd-LRP5-LRP6 complex that triggers β-catenin signaling. The first evidence supporting the role of *LRP6* in CRC predisposition was published by de Voer et al. (2016) [55]. Three predicted pathogenic missense variants were identified in individuals with a very early onset of the disease (<= 30 years). All the variants were located in β-propeller domains, which are involved in the binding of Wnt ligands and antagonists. Two of the three variants showed increased Wnt signaling activity in vitro. Despite additional predicted pathogenic variants being identified by Broderick et al. [9] and Belhadj et al. [10], no enrichment of likely pathogenic variants was observed in familial/early onset CRC patients compared to controls [10].

A truncating mutation in *SETD6*, a mono-methyltransferase that modulates Wnt and NF-kB signaling pathways, was identified in three CRC-affected siblings of an MMR-proficient Amsterdam I CRC family [79]. No disruptive or predicted pathogenic variants were identified in the 1006 CRC patients and 1609 controls studied by Chubb et al. [28].

Recently, likely pathogenic missense variants in *FAF1* were identified in two CRC families [80]. *FAF1* is a likely tumor suppressor gene that encodes a pro-apoptotic scaffolding protein that inhibits NF-κB nuclear translocation and activation, antagonizes the canonical Wnt signaling pathway, participates in DNA replication fork dynamics, and is involved in receptor-dependent and -independent apoptosis. Cosegregation results and functional analyses covering almost all the functions described led the authors to suggest that germline *FAF1* mutations are implicated in inherited susceptibility to CRC [80]. In contrast, exome sequencing data from cases and controls seem to suggest otherwise (Table S2) [28].

Predicted pathogenic germline variants in several components of the Wnt or TGF-Beta/BMP pathways, such as *CTBP2*, *WIF1*, *AXIN1, TWSG1*, and *BMP4* [71,77,81], have been identified in CRC patients. However, these findings are limited to the original study and thus, up to date, data is insufficient to get conclusions about their causal role in CRC predisposition. In the exome sequencing data evaluated by Chubb et al., except for *WIF1*, no over-representation of disruptive and/or predicted pathogenic variants in any of the mentioned genes was detected (Table S2) [28].

### 3.4. Additional Candidates

In addition to the candidate genes mentioned so far, at least 40 more have been proposed in the literature with different degrees of supporting evidence (Tables S1 and S2). In this section, we will briefly discuss only those that have been evaluated at least by two different groups.

*UNC5C*, a member of the family UNC5 of netrin receptors, was proposed as candidate gene for CRC predisposition [82] based on previous evidence demonstrating the role of UNC5C and other Netrin I receptors as tumor suppressors and their association with intestinal tumor initiation and progression [83,84]. After the identification of 5 carriers of novel or rare variants in 235 familial CRC probands, and based on the location of the predicted pathogenic variants, Coissieux et al. studied 4 exons in 582–984 CRC patients, finding 10 additional variants. Moreover, functional evidence supported the deleterious effect of p.Ala628Lys [82]. Küry et al. studied exon 11 in ~300 familial CRC patients and 300 unaffected controls, and genotyped p.Ala628Lys in a total of 1023 CRC patients and 821 controls, concluding that *UNC5C* germline pathogenic variants were extremely rare in CRC patients [85]. We performed a mutational screening of the whole gene and identified 8 rare or novel *UNC5C* variants in 529 unexplained CRC families and polyposis patients. Lack of association with CRC for at least 7 of the 8 identified variants was evident after cosegregation analyses and consideration of case-control data [86].

*SEMA4A*, a gene coding for the membrane-bound signaling protein Semaphorin 4A, was first associated with CRC predisposition by Schulz et al. (2014), who estimated a 6.8-fold increased CRC risk for the variant p.Pro682Ser [87]. Subsequently, Kinnersley et al. (2015) assessed the presence of p.Pro682Ser and p.Gly484Ala in ~7000 CRC cases and 10,000 controls, finding no association with CRC [88]. We performed a mutational screening of the gene in 473 familial/early onset CRC cases, finding one rare predicted pathogenic missense variant. Moreover, CRC case-control data showed no association for p.Pro682Ser. Finally, we performed a meta-analysis with all available data that showed a higher but not significant enrichment of predicted pathogenic variants in familial/early onset CRC cases compared to controls [10].

*LIMK2* encodes a Ser/Thr-protein kinase that plays an essential role in the regulation of actin filament dynamics and acts downstream of Rho family GTPase signal transduction, among other functions. Sequencing data from Chubb et al. (2016) identified 8 loss-of-function or canonical splice-site variants and one missense predicted pathogenic variant in 1006 cases, and one of them in 1609 controls. These included one recurrent frameshift variant, p.Gly574ArgfsTer12, detected in five cases and no controls [28]. We carried out the mutational screening of the gene in 473 familial/early onset CRC patients and identified two carriers of predicted pathogenic missense variants. Considering the two studies and comparing the results with gnomAD population data, we observed that disruptive and/or predicted pathogenic variants are not enriched in cases compared to controls [10]. 

*IL12RB1*, which causes immunodeficiency 30 (MIM# 614891) in an autosomal recessive, was also identified as a candidate gene by Chubb et al. (2016), after finding over-representation of germline loss-of-function variants in cases compared to controls [28]. Their findings were supported by a previous report of an immunodeficiency 30-affected family, where two heterozygous carriers had been diagnosed with gastric cancer in their 70s and a third carrier had developed three rectal tubular adenomas and two hyperplastic polyps by age 62 [89]. We identified three carriers of either disruptive or predicted pathogenic variants in 473 familial/early onset CRC patients [10]. When considering the two studies, we observed that *IL12RB1* disruptive variants are significantly enriched in familial/early onset CRC cases compared to controls [10].

*GALNT12*, which codes for N-acetylgalactosaminyltransferase-type 12, is highly expressed in the normal colon, is downregulated in a significant proportion of CRCs [90,91], and is located (9q21-33) in close proximity to the linkage peak 9q22-31, recurrently found when studying familial CRC cases [92,93,94,95], making it an especially attractive candidate gene for CRC predisposition. Guda et al. (2009) performed a mutational screening of *GALNT12* in 272 CRC patients and 192 cancer-free controls, finding rare *GALNT12* germline variants in 7 CRC cases and no controls [96]. Clarke et al. (2012) reported the presence of two functionally relevant deleterious variants in 4 of 118 familial CRC patients, with none among the 26 probands who met the Amsterdam I criteria [97]. We assessed the status of the gene in 103 Amsterdam-positive CRC families. Despite the identification of 18 rare variants, none seemed to be functionally relevant [98]. In this line, sequencing data obtained by Chubb et al. showed an absence of loss-of-function variants among 1006 familial/earlyonset CRC patients, and no over-representation of predicted pathogenic variants in the cases (8/1006) compared to controls (21/1609) [28].

*PTPN12*, a regulator of cell motility, was identified as a candidate gene for CRC predisposition when 3 novel or rare germline variants affecting highly conserved amino acids were identified in 3 out of 55 early onset CRC patients studied by exome sequencing. When the gene was studied in 174 additional early onset CRC patients, the authors identified one extra carrier [55]. Data from the exomes analyzed by Chubb et al. revealed 6 predicted pathogenic variants among the 1006 (0.6%) familial/early onset CRC patients and 5 predicted pathogenic variants, including one loss-of-function, among the 1609 controls (0.3%) [9,28]. The evidence gathered is still insufficient to consider *PTPN12* as a hereditary CRC gene, thus requiring the analysis of additional cohorts in order to provide a definitive answer about *PTPN12*’s role in CRC predisposition.

A 170-kb head-to-tail duplication upstream of *PTPRJ* that causes the silencing of the gene as a result of the hypermethylation of its promoter was identified in a CRC patient included in a cohort of 40 patients diagnosed with MMR-proficient early onset CRC. While no *PTPRJ* copy number variants (CNVs) were found in > 2650 cancer-free controls, the screening of an additional cohort of ~1500 CRC patients detected a 564-kb duplication in a 39-year-old CRC patient, also causing *PTPRJ* promoter methylation [99]. We recently investigated the presence of constitutional *PTPRJ* promoter methylation in 473 familial CRC, finding no epigenetic changes [10], and thus supporting the rarity of this type of alteration (2 carriers identified among ~2000 CRC patients tested). 

Germline large deletions or CNVs and rare missense variants in the tumor suppressor gene *FOCAD* (focadhesin) have been reported in early onset and familial CRC patients; however, whether those cause or do not cause increased CRC (or polyposis) risk remains an unsolved issue [10,59,100,101].

## 4. Non-CRC Hereditary Cancer Genes

The use of next-generation sequencing-based approaches, including exome- or genome-sequencing and multi-gene panels, either for the discovery of new candidate genes or for genetic diagnosis in CRC patients, has allowed the identification of germline pathogenic variants in hereditary cancer genes a priori not associated with increased CRC risk, or at least not with the colonic phenotype observed in the carrier. Whether these are the actual cause of the increased risk observed or a representation of the background population frequency of those gene variants remains a matter of controversy. The contribution of non-CRC cancer-predisposing genes to undefined familial/early onset CRC may reach 3–7% [28,102]. In this section, the involvement of *BRCA1*, *BRCA2*, and *TP53* genes will be briefly discussed. 

Two of the most frequently altered non-CRC hereditary cancer genes found in CRC patients are *BRCA1* and *BRCA2*; however, the debate about whether those actually increase the risk of CRC is still ongoing [61,103,104,105,106,107,108]. A recent meta-analysis, together with familial/early onset CRC case-control data, indicate that *BRCA1* and *BRCA2* pathogenic variants do not increase the risk to CRC [109,110]. Contrarily, another meta-analysis suggests that pathogenic variants in *BRCA1* increase CRC risk (OR = 1.56) but not in *BRCA2* [111]. Another gene that deserves attention in this section is *TP53*, where pathogenic variants have been recurrently found in familial/early onset and unselected CRC patients [3,4,5,6,28,32,102,112,113,114,115]. As occurs with other non-CRC genes, the debate about *TP53*’s causal role in CRC predisposition is open for discussion. While some studies show no significant association [110], the most recent surveillance guidelines for Li–Fraumeni patients include CRC screening [116,117]. 

Moreover, germline pathogenic variants in genes associated with different colorectal phenotypes have also been identified in MMR-proficient nonpolyposis CRC patients. Such is the case for several polyposis-associated genes, including *MUTYH*, *BMPR1A* or *POLE*, and *POLD1*, among others [118,119,120,121,122,123,124,125]. For details on other hereditary cancer genes, you may read Valle et al. (2019) [126] and look up the frequency of rare predicted pathogenic variants in familial/early onset CRC patients and controls in Table S2 [28].

## 5. Conclusions/Final Remarks

**Figure 2:**
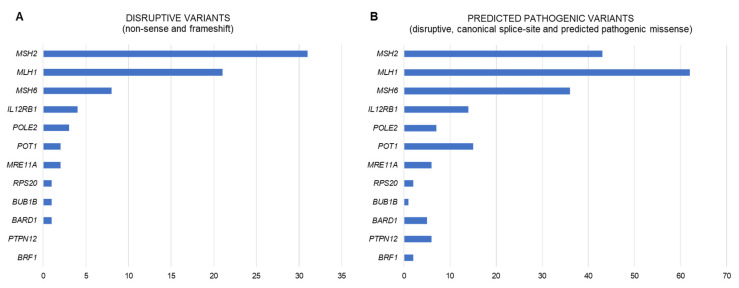
(**A**) Number of disruptive (non-sense and frameshift) and (**B**) predicted pathogenic (disruptive, canonical splice-site, and predicted pathogenic missense) variants identified in the MMR genes *MLH1*, *MSH2* and *MSH6*, *RPS20*, and in the most promising candidate genes for nonpolyposis CRC predisposition, in 1006 familial/early onset CRC cases. *PMS2* was not included in the graph due to the possibility of miscalled variants due to the existence of multiple pseudogenes. Data obtained from Chubb et al., 2016 [28].

Despite the enormous efforts made to identify the genetic cause of familial/early onset MMR-proficient nonpolyposis CRC, the contribution of the identified candidate genes, if any, is extremely small, almost negligible. To date, only pathogenic variants in *RPS20* may be convincedly linked to hereditary CRC. Nevertheless, other genes, such as *MRE11*, *BARD1*, *POT1*, *BUB1B*, *POLE2*, *BRF1*, *IL12RB1*, *PTPN12*, or the epigenetic alteration of *PTPRJ*, show promising evidence that supports their involvement in CRC predisposition (Figure 1). Additional studies are needed to finally confirm (or discard) their causal role as hereditary CRC genes and if so, define the associated cancer risks and tumor spectra. However, even if these associations are confirmed, the proportion of cases explained by alterations in these genes is very low (Figure 2). Disruptive (loss of function) variants in all of the most promising candidate genes mentioned above are found in 1.3% (13/1006) of familial/early onset CRC patients, and reach 5.5% when considering disruptive, canonical splice-site, and predicted pathogenic missense variants (Table S3; data source: [28]).

Other factors might explain the missing heritability in CRC. Promising results have recently been obtained when testing the hypothesis that the accumulation of low-risk CRC alleles may explain a subset of early onset and familial CRC cases [127]. Shared environmental exposures, gene–environmental interactions, or oligogenic inheritance of moderate/low-risk alleles might also contribute to the aggregation of CRC in these families and/or to the early age of onset.

## Supplementary Materials

The following are available online at https://www.mdpi.com/2077-0383/9/6/1954/s1, Table S1: Candidate genes proposed for CRC predisposition, molecular pathways where they are involved and relevant bibliography, Table S2: Number of disruptive (non-sense and frameshift) and predicted pathogenic (disruptive, canonical splice-site and predicted pathogenic missense) variants identified in 1006 familial/early-onset cases and 1609 controls (Data obtained from Chubb et al., 2016 [28]), Table S3: Number of disruptive and predicted pathogenic variants in the most promising CRC candidates identified in 1006 familial/early-onset CRC patients and 1609 (Data obtained from from Chubb et al., 2016 [28]).

## References

[1] Frank C., Fallah M., Sundquist J., Hemminki A., Hemminki K. (2015). Population Landscape of Familial Cancer. Sci. Rep..

[2] Frank C., Sundquist J., Yu H., Hemminki A., Hemminki K. (2017). Concordant and discordant familial cancer: Familial risks, proportions and population impact. Int. J. Cancer.

[3] DeRycke M.S., Gunawardena S., Balcom J.R., Pickart A.M., Waltman L.A., French A.J., McDonnell S., Riska S.M., Fogarty Z.C., Larson M.C. (2017). Targeted sequencing of 36 known or putative colorectal cancer susceptibility genes. Mol. Genet. Genom. Med..

[4] Yurgelun M.B., Kulke M.H., Fuchs C.S., Allen B.A., Uno H., Hornick J.L., Ukaegbu C.I., Brais L.K., McNamara P.G., Mayer R.J. (2017). Cancer Susceptibility Gene Mutations in Individuals With Colorectal Cancer. J. Clin. Oncol..

[5] AlDubayan S.H., Giannakis M., Moore N.D., Han G.C., Reardon B., Hamada T., Mu X.J., Nishihara R., Qian Z., Liu L. (2018). Inherited DNA-Repair Defects in Colorectal Cancer. Am. J. Hum. Genet..

[6] You Y.N., Borras E., Chang K., Price B.A., Mork M., Chang G.J., Rodriguez-Bigas M.A., Bednarski B.K., Meric-Bernstam F., Vilar E. (2019). Detection of Pathogenic Germline Variants Among Patients With Advanced Colorectal Cancer Undergoing Tumor Genomic Profiling for Precision Medicine. Dis. Colon Rectum.

[7] Valle L., Valle L., Gruber S.B., Capellá G. (2018). Mismatch Repair-Proficient Hereditary Nonpolyposis Colorectal Cancer. Hereditary Colorectal Cancer: Genetic Basis and Clinical Implications.

[8] Nieminen T.T., O’Donohue M.F., Wu Y., Lohi H., Scherer S.W., Paterson A.D., Ellonen P., Abdel-Rahman W.M., Valo S., Mecklin J.P. (2014). Germline mutation of RPS20, encoding a ribosomal protein, causes predisposition to hereditary nonpolyposis colorectal carcinoma without DNA mismatch repair deficiency. Gastroenterology.

[9] Broderick P., Dobbins S.E., Chubb D., Kinnersley B., Dunlop M.G., Tomlinson I., Houlston R.S. (2017). Validation of Recently Proposed Colorectal Cancer Susceptibility Gene Variants in an Analysis of Families and Patients—A Systematic Review. Gastroenterology.

[10] Belhadj S., Terradas M., Munoz-Torres P.M., Aiza G., Navarro M., Capellá G., Valle L. (2020). Candidate genes for hereditary colorectal cancer: Mutational screening and systematic review. Hum. Mutat..

[11] Thompson B.A., Snow A.K., Koptiuch C., Kohlmann W.K., Mooney R., Johnson S., Huff C.D., Yu Y., Teerlink C.C., Feng B.J. (2020). A novel ribosomal protein S20 variant in a family with unexplained colorectal cancer and polyposis. Clin. Genet..

[12] Arora S., Yan H., Cho I., Fan H.Y., Luo B., Gai X., Bodian D.L., Vockley J.G., Zhou Y., Handorf E.A. (2015). Genetic Variants That Predispose to DNA Double-Strand Breaks in Lymphocytes From a Subset of Patients With Familial Colorectal Carcinomas. Gastroenterology.

[13] Esteban-Jurado C., Vila-Casadesus M., Garre P., Lozano J.J., Pristoupilova A., Beltran S., Munoz J., Ocana T., Balaguer F., Lopez-Ceron M. (2015). Whole-exome sequencing identifies rare pathogenic variants in new predisposition genes for familial colorectal cancer. Genet. Med..

[14] Reilly N.M., Novara L., Di Nicolantonio F., Bardelli A. (2019). Exploiting DNA repair defects in colorectal cancer. Mol. Oncol..

[15] Kurz E.U., Lees-Miller S.P. (2004). DNA damage-induced activation of ATM and ATM-dependent signaling pathways. DNA Repair.

[16] Di Leonardo A., Linke S.P., Clarkin K., Wahl G.M. (1994). DNA damage triggers a prolonged p53-dependent G1 arrest and long-term induction of Cip1 in normal human fibroblasts. Genes Dev..

[17] Bakkenist C.J., Kastan M.B. (2004). Initiating cellular stress responses. Cell.

[18] Kastan M.B., Onyekwere O., Sidransky D., Vogelstein B., Craig R.W. (1991). Participation of p53 protein in the cellular response to DNA damage. Cancer Res..

[19] Hanahan D., Weinberg R.A. (2011). Hallmarks of cancer: The next generation. Cell.

[20] Al-Tassan N., Chmiel N.H., Maynard J., Fleming N., Livingston A.L., Williams G.T., Hodges A.K., Davies D.R., David S.S., Sampson J.R. (2002). Inherited variants of MYH associated with somatic G:C→T:A mutations in colorectal tumors. Nat. Genet..

[21] Weren R.D., Ligtenberg M.J., Kets C.M., de Voer R.M., Verwiel E.T., Spruijt L., van Zelst-Stams W.A., Jongmans M.C., Gilissen C., Hehir-Kwa J.Y. (2015). A germline homozygous mutation in the base-excision repair gene NTHL1 causes adenomatous polyposis and colorectal cancer. Nat. Genet..

[22] Kim I.J., Ku J.L., Kang H.C., Park J.H., Yoon K.A., Shin Y., Park H.W., Jang S.G., Lim S.K., Han S.Y. (2004). Mutational analysis of OGG1, MYH, MTH1 in FAP, HNPCC and sporadic colorectal cancer patients: R154H OGG1 polymorphism is associated with sporadic colorectal cancer patients. Hum. Genet..

[23] Farrington S.M., Tenesa A., Barnetson R., Wiltshire A., Prendergast J., Porteous M., Campbell H., Dunlop M.G. (2005). Germline susceptibility to colorectal cancer due to base-excision repair gene defects. Am. J. Hum. Genet..

[24] Garre P., Briceno V., Xicola R.M., Doyle B.J., de la Hoya M., Sanz J., Llovet P., Pescador P., Puente J., Diaz-Rubio E. (2011). Analysis of the oxidative damage repair genes NUDT1, OGG1, and MUTYH in patients from mismatch repair proficient HNPCC families (MSS-HNPCC). Clin. Cancer Res..

[25] Morak M., Massdorf T., Sykora H., Kerscher M., Holinski-Feder E. (2011). First evidence for digenic inheritance in hereditary colorectal cancer by mutations in the base excision repair genes. Eur. J. Cancer.

[26] Smith C.G., West H., Harris R., Idziaszczyk S., Maughan T.S., Kaplan R., Richman S., Quirke P., Seymour M., Moskvina V. (2013). Role of the oxidative DNA damage repair gene OGG1 in colorectal tumorigenesis. J. Natl. Cancer Inst..

[27] Kinnersley B., Buch S., Castellvi-Bel S., Farrington S.M., Forsti A., Hampe J., Hemminki K., Hofstra R.M., Northwood E., Palles C. (2014). Re: Role of the oxidative DNA damage repair gene OGG1 in colorectal tumorigenesis. J. Natl. Cancer Inst..

[28] Chubb D., Broderick P., Dobbins S.E., Frampton M., Kinnersley B., Penegar S., Price A., Ma Y.P., Sherborne A.L., Palles C. (2016). Rare disruptive mutations and their contribution to the heritable risk of colorectal cancer. Nat. Commun..

[29] Mur P., Jemth A.S., Bevc L., Amaral N., Navarro M., Valdés-Mas R., Pons T., Aiza G., Urioste M., Valencia A. (2018). Germline variation in the oxidative DNA repair genes NUDT1 and OGG1 is not associated with hereditary colorectal cancer or polyposis. Hum. Mutat..

[30] Broderick P., Bagratuni T., Vijayakrishnan J., Lubbe S., Chandler I., Houlston R.S. (2006). Evaluation of NTHL1, NEIL1, NEIL2, MPG, TDG, UNG and SMUG1 genes in familial colorectal cancer predisposition. BMC Cancer.

[31] Dallosso A.R., Dolwani S., Jones N., Jones S., Colley J., Maynard J., Idziaszczyk S., Humphreys V., Arnold J., Donaldson A. (2008). Inherited predisposition to colorectal adenomas caused by multiple rare alleles of MUTYH but not OGG1, NUDT1, NTH1 or NEIL 1, 2 or 3. Gut.

[32] Martin-Morales L., Rofes P., Diaz-Rubio E., Llovet P., Lorca V., Bando I., Perez-Segura P., de la Hoya M., Garre P., Garcia-Barberan V. (2018). Novel genetic mutations detected by multigene panel are associated with hereditary colorectal cancer predisposition. PLoS ONE.

[33] Díaz-Gay M., Franch-Expósito S., Arnau-Collell C., Park S., Supek F., Muñoz J., Bonjoch L., Gratacós-Mulleras A., Sánchez-Rojas P.A., Esteban-Jurado C. (2019). Integrated Analysis of Germline and Tumor DNA Identifies New Candidate Genes Involved in Familial Colorectal Cancer. Cancer.

[34] Lind G.E., Thorstensen L., Løvig T., Meling G.I., Hamelin R., Rognum T.O., Esteller M., Lothe R.A. (2004). A CpG island hypermethylation profile of primary colorectal carcinomas and colon cancer cell lines. Mol. Cancer.

[35] Shen L., Kondo Y., Rosner G.L., Xiao L., Hernandez N.S., Vilaythong J., Houlihan P.S., Krouse R.S., Prasad A.R., Einspahr J.G. (2005). MGMT promoter methylation and field defect in sporadic colorectal cancer. J. Natl. Cancer Inst..

[36] Belhadj S., Moutinho C., Mur P., Setien F., Llinàs-Arias P., Pérez-Salvia M., Pons T., Pineda M., Brunet J., Navarro M. (2019). Germline variation in O6-Methylguanine-DNA Methyltransferase (MGMT) as cause of hereditary colorectal cancer. Cancer Lett..

[37] Gisselsson D., Pettersson L., Höglund M., Heidenblad M., Gorunova L., Wiegant J., Mertens F., Dal Cin P., Mitelman F., Mandahl N. (2000). Chromosomal breakage-fusion-bridge events cause genetic intratumor heterogeneity. Proc. Natl. Acad. Sci. USA..

[38] Abdel-Rahman W.M., Ollikainen M., Kariola R., Järvinen H.J., Mecklin J.P., Nyström-Lahti M., Knuutila S., Peltomäki P. (2005). Comprehensive characterization of HNPCC-related colorectal cancers reveals striking molecular features in families with no germline mismatch repair gene mutations. Oncogene.

[39] Bellido F., Pineda M., Sanz-Pamplona R., Navarro M., Nadal M., Lázaro C., Blanco I., Moreno V., Capellá G., Valle L. (2014). Comprehensive molecular characterisation of hereditary non-polyposis colorectal tumours with mismatch repair proficiency. Eur. J. Cancer.

[40] Ihara K., Yamaguchi S., Ueno N., Tani Y., Shida Y., Ogata H., Domeki Y., Okamoto K., Nakajima M., Sasaki K. (2016). Expression of DNA double-strand break repair proteins predicts the response and prognosis of colorectal cancer patients undergoing oxaliplatin-based chemotherapy. Oncol. Rep..

[41] Situ Y., Chung L., Lee C.S., Ho V. (2019). MRN (MRE11-RAD50-NBS1) Complex in Human Cancer and Prognostic Implications in Colorectal Cancer. Int. J. Mol. Sci..

[42] Gruber S.B., Ellis N.A., Scott K.K., Almog R., Kolachana P., Bonner J.D., Kirchhoff T., Tomsho L.P., Nafa K., Pierce H. (2002). BLM heterozygosity and the risk of colorectal cancer. Science.

[43] Sokolenko A.P., Iyevleva A.G., Preobrazhenskaya E.V., Mitiushkina N.V., Abysheva S.N., Suspitsin E.N., Kuligina E.S., Gorodnova T.V., Pfeifer W., Togo A.V. (2012). High prevalence and breast cancer predisposing role of the BLM c.1642 C>T (Q548X) mutation in Russia. Int. J. Cancer.

[44] Thompson E.R., Doyle M.A., Ryland G.L., Rowley S.M., Choong D.Y., Tothill R.W., Thorne H., Barnes D.R., Li J., Ellul J. (2012). Exome sequencing identifies rare deleterious mutations in DNA repair genes FANCC and BLM as potential breast cancer susceptibility alleles. PLoS Genet..

[45] Prokofyeva D., Bogdanova N., Dubrowinskaja N., Bermisheva M., Takhirova Z., Antonenkova N., Turmanov N., Datsyuk I., Gantsev S., Christiansen H. (2013). Nonsense mutation p.Q548X in BLM, the gene mutated in Bloom’s syndrome, is associated with breast cancer in Slavic populations. Breast Cancer Res. Treat..

[46] De Voer R.M., Hahn M.M., Mensenkamp A.R., Hoischen A., Gilissen C., Henkes A., Spruijt L., van Zelst-Stams W.A., Kets C.M., Verwiel E.T. (2015). Deleterious Germline BLM Mutations and the Risk for Early-onset Colorectal Cancer. Sci. Rep..

[47] Novak E.M., Halley N.S., Gimenez T.M., Rangel-Santos A., Azambuja A.M., Brumatti M., Pereira P.L., Vince C.S., Giorgi R.R., Bendit I. (2016). BLM germline and somatic PKMYT1 and AHCY mutations: Genetic variations beyond MYCN and prognosis in neuroblastoma. Med. Hypotheses.

[48] Raskin L., Guo Y., Du L., Clendenning M., Rosty C., Lindor N.M., Gruber S.B., Buchanan D.D., Colon Cancer Family Registry (CCFR) (2017). Targeted sequencing of established and candidate colorectal cancer genes in the Colon Cancer Family Registry Cohort. Oncotarget.

[49] Schayek H., Laitman Y., Katz L.H., Pras E., Ries-Levavi L., Barak F., Friedman E. (2017). Colorectal and Endometrial Cancer Risk and Age at Diagnosis in BLMAsh Mutation Carriers. Isr. Med. Assoc. J..

[50] Walker L.C., Pearson J.F., Wiggins G.A., Giles G.G., Hopper J.L., Southey M.C. (2017). Increased genomic burden of germline copy number variants is associated with early onset breast cancer: Australian breast cancer family registry. Breast Cancer Res..

[51] Cleary S.P., Zhang W., Di Nicola N., Aronson M., Aube J., Steinman A., Haddad R., Redston M., Gallinger S., Narod S.A. (2003). Heterozygosity for the BLM(Ash) mutation and cancer risk. Cancer Res..

[52] Baris H.N., Kedar I., Halpern G.J., Shohat T., Magal N., Ludman M.D., Shohat M. (2007). Prevalence of breast and colorectal cancer in Ashkenazi Jewish carriers of Fanconi anemia and Bloom syndrome. Isr. Med. Assoc. J..

[53] Antczak A., Kluźniak W., Wokołorczyk D., Kashyap A., Jakubowska A., Gronwald J., Huzarski T., Byrski T., Dębniak T., Masojć B. (2013). A common nonsense mutation of the BLM gene and prostate cancer risk and survival. Gene.

[54] Laitman Y., Boker-Keinan L., Berkenstadt M., Liphsitz I., Weissglas-Volkov D., Ries-Levavi L., Sarouk I., Pras E., Friedman E. (2016). The risk for developing cancer in Israeli ATM, BLM, and FANCC heterozygous mutation carriers. Cancer Genet..

[55] De Voer R.M., Hahn M.M., Weren R.D., Mensenkamp A.R., Gilissen C., van Zelst-Stams W.A., Spruijt L., Kets C.M., Zhang J., Venselaar H. (2016). Identification of Novel Candidate Genes for Early-Onset Colorectal Cancer Susceptibility. PLoS Genet..

[56] Bellido F., Sowada N., Mur P., Lazaro C., Pons T., Valdes-Mas R., Pineda M., Aiza G., Iglesias S., Soto J.L. (2018). Association Between Germline Mutations in BRF1, a Subunit of the RNA Polymerase III Transcription Complex, and Hereditary Colorectal Cancer. Gastroenterology.

[57] Goldberg Y., Halpern N., Hubert A., Adler S.N., Cohen S., Plesser-Duvdevani M., Pappo O., Shaag A., Meiner V. (2015). Mutated MCM9 is associated with predisposition to hereditary mixed polyposis and colorectal cancer in addition to primary ovarian failure. Cancer Genet..

[58] Terradas M., Munoz-Torres P.M., Belhadj S., Aiza G., Navarro M., Brunet J., Capellá G., Valle L. (2019). Contribution to colonic polyposis of recently proposed predisposing genes and assessment of the prevalence of NTHL1- and MSH3-associated polyposes. Hum. Mutat..

[59] Ciavarella M., Miccoli S., Prossomariti A., Pippucci T., Bonora E., Buscherini F., Palombo F., Zuntini R., Balbi T., Ceccarelli C. (2018). Somatic APC mosaicism and oligogenic inheritance in genetically unsolved colorectal adenomatous polyposis patients. Eur. J. Hum. Genet..

[60] Garre P., Martin L., Sanz J., Romero A., Tosar A., Bando I., Llovet P., Diaque P., Garcia-Paredes B., Diaz-Rubio E. (2015). BRCA2 gene: A candidate for clinical testing in familial colorectal cancer type X. Clin. Genet..

[61] Esteban-Jurado C., Franch-Exposito S., Munoz J., Ocana T., Carballal S., Lopez-Ceron M., Cuatrecasas M., Vila-Casadesus M., Lozano J.J., Serra E. (2016). The Fanconi anemia DNA damage repair pathway in the spotlight for germline predisposition to colorectal cancer. Eur. J. Hum. Genet..

[62] Calvete O., Martinez P., Garcia-Pavia P., Benitez-Buelga C., Paumard-Hernández B., Fernandez V., Dominguez F., Salas C., Romero-Laorden N., Garcia-Donas J. (2015). A mutation in the POT1 gene is responsible for cardiac angiosarcoma in TP53-negative Li-Fraumeni-like families. Nat. Commun..

[63] Calvete O., Garcia-Pavia P., Domínguez F., Bougeard G., Kunze K., Braeuninger A., Teule A., Lasa A., Ramón Y., Cajal T. (2017). The wide spectrum of POT1 gene variants correlates with multiple cancer types. Eur. J. Hum. Genet..

[64] Bainbridge M.N., Armstrong G.N., Gramatges M.M., Bertuch A.A., Jhangiani S.N., Doddapaneni H., Lewis L., Tombrello J., Tsavachidis S., Liu Y. (2015). Germline mutations in shelterin complex genes are associated with familial glioma. J. Natl. Cancer Inst..

[65] Müller C., Krunic M., Wendt J., von Haeseler A., Okamoto I. (2018). Germline Variants in the POT1-Gene in High-Risk Melanoma Patients in Austria. G3 (Bethesda).

[66] McMaster M.L., Sun C., Landi M.T., Savage S.A., Rotunno M., Yang X.R., Jones K., Vogt A., Hutchinson A., Zhu B. (2018). Germline mutations in Protection of Telomeres 1 in two families with Hodgkin lymphoma. Br. J. Haematol..

[67] Speedy H.E., Kinnersley B., Chubb D., Broderick P., Law P.J., Litchfield K., Jayne S., Dyer M.J.S., Dearden C., Follows G.A. (2016). Germ line mutations in shelterin complex genes are associated with familial chronic lymphocytic leukemia. Blood.

[68] De Voer R.M., Geurts van Kessel A., Weren R.D., Ligtenberg M.J., Smeets D., Fu L., Vreede L., Kamping E.J., Verwiel E.T., Hahn M.M. (2013). Germline mutations in the spindle assembly checkpoint genes BUB1 and BUB3 are risk factors for colorectal cancer. Gastroenterology.

[69] Mur P., De Voer R.M., Olivera-Salguero R., Rodriguez-Perales S., Pons T., Setien F., Aiza G., Valdes-Mas R., Bertini A., Pineda M. (2018). Germline mutations in the spindle assembly checkpoint genes BUB1 and BUB3 are infrequent in familial colorectal cancer and polyposis. Mol. Cancer.

[70] Rio Frio T., Lavoie J., Hamel N., Geyer F.C., Kushner Y.B., Novak D.J., Wark L., Capelli C., Reis-Filho J.S., Mai S. (2010). Homozygous BUB1B mutation and susceptibility to gastrointestinal neoplasia. N. Engl. J. Med..

[71] DeRycke M.S., Gunawardena S.R., Middha S., Asmann Y.W., Schaid D.J., McDonnell S.K., Riska S.M., Eckloff B.W., Cunningham J.M., Fridley B.L. (2013). Identification of novel variants in colorectal cancer families by high-throughput exome sequencing. Cancer Epidemiol. Biomark. Prev..

[72] Hahn M.M., Vreede L., Bemelmans S.A., van der Looij E., van Kessel A.G., Schackert H.K., Ligtenberg M.J., Hoogerbrugge N., Kuiper R.P., de Voer R.M. (2016). Prevalence of germline mutations in the spindle assembly checkpoint gene BUB1B in individuals with early-onset colorectal cancer. Genes Chromosomes Cancer.

[73] Cicek M.S., Cunningham J.M., Fridley B.L., Serie D.J., Bamlet W.R., Diergaarde B., Haile R.W., Le Marchand L., Krontiris T.G., Younghusband H.B. (2012). Colorectal cancer linkage on chromosomes 4q21, 8q13, 12q24, and 15q22. PLoS ONE.

[74] Tanskanen T., Gylfe A.E., Katainen R., Taipale M., Renkonen-Sinisalo L., Jarvinen H., Mecklin J.P., Bohm J., Kilpivaara O., Pitkanen E. (2015). Systematic search for rare variants in Finnish early-onset colorectal cancer patients. Cancer Genet..

[75] Spier I., Holzapfel S., Altmuller J., Zhao B., Horpaopan S., Vogt S., Chen S., Morak M., Raeder S., Kayser K. (2015). Frequency and phenotypic spectrum of germline mutations in POLE and seven other polymerase genes in 266 patients with colorectal adenomas and carcinomas. Int. J. Cancer.

[76] Rogers R.F., Walton M.I., Cherry D.L., Collins I., Clarke P.A., Garrett M.D., Workman P. (2020). CHK1 Inhibition Is Synthetically Lethal with Loss of B-Family DNA Polymerase Function in Human Lung and Colorectal Cancer Cells. Cancer Res..

[77] Gylfe A.E., Katainen R., Kondelin J., Tanskanen T., Cajuso T., Hanninen U., Taipale J., Taipale M., Renkonen-Sinisalo L., Jarvinen H. (2013). Eleven candidate susceptibility genes for common familial colorectal cancer. PLoS Genet..

[78] Valle L., Vilar E., Tavtigian S.V., Stoffel E.M. (2019). Genetic predisposition to colorectal cancer: Syndromes, genes, classification of genetic variants and implications for precision medicine. J. Pathol..

[79] Martin-Morales L., Feldman M., Vershinin Z., Garre P., Caldes T., Levy D. (2017). SETD6 dominant negative mutation in familial colorectal cancer type X. Hum. Mol. Genet..

[80] Bonjoch L., Franch-Expósito S., Garre P., Belhadj S., Muñoz J., Arnau-Collell C., Díaz-Gay M., Gratacós-Mulleras A., Raimondi G., Esteban-Jurado C. (2020). GERMLINE MUTATIONS IN FAF1 ARE ASSOCIATED WITH HEREDITARY COLORECTAL CANCER. Gastroenterology.

[81] Wei C., Peng B., Han Y., Chen W.V., Rother J., Tomlinson G.E., Boland C.R., Chaussabel D., Frazier M.L., Amos C.I. (2015). Mutations of HNRNPA0 and WIF1 predispose members of a large family to multiple cancers. Fam. Cancer.

[82] Coissieux M.M., Tomsic J., Castets M., Hampel H., Tuupanen S., Andrieu N., Comeras I., Drouet Y., Lasset C., Liyanarachchi S. (2011). Variants in the netrin-1 receptor UNC5C prevent apoptosis and increase risk of familial colorectal cancer. Gastroenterology.

[83] Grady W.M. (2007). Making the case for DCC and UNC5C as tumor-suppressor genes in the colon. Gastroenterology.

[84] Mazelin L., Bernet A., Bonod-Bidaud C., Pays L., Arnaud S., Gespach C., Bredesen D.E., Scoazec J.Y., Mehlen P. (2004). Netrin-1 controls colorectal tumorigenesis by regulating apoptosis. Nature.

[85] Küry S., Garrec C., Airaud F., Breheret F., Guibert V., Frenard C., Jiao S., Bonneau D., Berthet P., Bossard C. (2014). Evaluation of the colorectal cancer risk conferred by rare UNC5C alleles. World J. Gastroenterol..

[86] Mur P., Elena S.C., Ausso S., Aiza G., Rafael V.M., Pineda M., Navarro M., Brunet J., Urioste M., Lazaro C. (2016). Scarce evidence of the causal role of germline mutations in UNC5C in hereditary colorectal cancer and polyposis. Sci. Rep..

[87] Schulz E., Klampfl P., Holzapfel S., Janecke A.R., Ulz P., Renner W., Kashofer K., Nojima S., Leitner A., Zebisch A. (2004). Germline variants in the SEMA4A gene predispose to familial colorectal cancer type X. Nat. Commun..

[88] Kinnersley B., Chubb D., Dobbins S.E., Frampton M., Buch S., Timofeeva M.N., Castellvi-Bel S., Farrington S.M., Forsti A., Hampe J. (2016). Correspondence: SEMA4A variation and risk of colorectal cancer. Nat. Commun..

[89] Vogelaar I.P., van der Post R.S., van de Vosse E., van Krieken J.H., Hoogerbrugge N., Ligtenberg M.J., Gómez García E. (2015). Gastric cancer in three relatives of a patient with a biallelic IL12RB1 mutation. Fam. Cancer.

[90] Guo J.M., Zhang Y., Cheng L., Iwasaki H., Wang H., Kubota T., Tachibana K., Narimatsu H. (2002). Molecular cloning and characterization of a novel member of the UDP-GalNAc:polypeptide N-acetylgalactosaminyltransferase family, pp-GalNAc-T12. FEBS Lett..

[91] Guo J.M., Chen H.L., Wang G.M., Zhang Y.K., Narimatsu H. (2004). Expression of UDP-GalNAc:polypeptide N-acetylgalactosaminyltransferase-12 in gastric and colonic cancer cell lines and in human colorectal cancer. Oncology.

[92] Wiesner G.L., Daley D., Lewis S., Ticknor C., Platzer P., Lutterbaugh J., MacMillen M., Baliner B., Willis J., Elston R.C. (2003). A subset of familial colorectal neoplasia kindreds linked to chromosome 9q22.2-31.2. Proc. Natl. Acad. Sci. USA.

[93] Skoglund J., Djureinovic T., Zhou X.L., Vandrovcova J., Renkonen E., Iselius L., Bisgaard M.L., Peltomaki P., Lindblom A. (2006). Linkage analysis in a large Swedish family supports the presence of a susceptibility locus for adenoma and colorectal cancer on chromosome 9q22.32-31.1. J. Med. Genet..

[94] Kemp Z.E., Carvajal-Carmona L.G., Barclay E., Gorman M., Martin L., Wood W., Rowan A., Donohue C., Spain S., Jaeger E. (2006). Evidence of linkage to chromosome 9q22.33 in colorectal cancer kindreds from the United Kingdom. Cancer Res..

[95] Gray-McGuire C., Guda K., Adrianto I., Lin C.P., Natale L., Potter J.D., Newcomb P., Poole E.M., Ulrich C.M., Lindor N. (2010). Confirmation of linkage to and localization of familial colon cancer risk haplotype on chromosome 9q22. Cancer Res..

[96] Guda K., Moinova H., He J., Jamison O., Ravi L., Natale L., Lutterbaugh J., Lawrence E., Lewis S., Willson J.K. (2009). Inactivating germ-line and somatic mutations in polypeptide N-acetylgalactosaminyltransferase 12 in human colon cancers. Proc. Natl. Acad. Sci. USA..

[97] Clarke E., Green R.C., Green J.S., Mahoney K., Parfrey P.S., Younghusband H.B., Woods M.O. (2012). Inherited deleterious variants in GALNT12 are associated with CRC susceptibility. Hum. Mutat..

[98] Segui N., Pineda M., Navarro M., Lazaro C., Brunet J., Infante M., Duran M., Soto J.L., Blanco I., Capella G. (2014). GALNT12 is not a major contributor of familial colorectal cancer type X. Hum. Mutat..

[99] Venkatachalam R., Ligtenberg M.J., Hoogerbrugge N., Schackert H.K., Gorgens H., Hahn M.M., Kamping E.J., Vreede L., Hoenselaar E., van der Looij E. (2010). Germline epigenetic silencing of the tumor suppressor gene PTPRJ in early-onset familial colorectal cancer. Gastroenterology.

[100] Venkatachalam R., Verwiel E.T., Kamping E.J., Hoenselaar E., Gorgens H., Schackert H.K., van Krieken J.H., Ligtenberg M.J., Hoogerbrugge N., van Kessel A.G. (2011). Identification of candidate predisposing copy number variants in familial and early-onset colorectal cancer patients. Int. J. Cancer.

[101] Weren R.D., Venkatachalam R., Cazier J.B., Farin H.F., Kets C.M., de Voer R.M., Vreede L., Verwiel E.T., van Asseldonk M., Kamping E.J. (2015). Germline deletions in the tumour suppressor gene FOCAD are associated with polyposis and colorectal cancer development. J. Pathol..

[102] Pearlman R., Frankel W.L., Swanson B., Zhao W., Yilmaz A., Miller K., Bacher J., Bigley C., Nelsen L., Goodfellow P.J. (2017). Prevalence and Spectrum of Germline Cancer Susceptibility Gene Mutations Among Patients With Early-Onset Colorectal Cancer. JAMA Oncol..

[103] Kirchhoff T., Satagopan J.M., Kauff N.D., Huang H., Kolachana P., Palmer C., Rapaport H., Nafa K., Ellis N.A., Offit K. (2004). Frequency of BRCA1 and BRCA2 mutations in unselected Ashkenazi Jewish patients with colorectal cancer. J. Natl. Cancer Inst..

[104] Niell B.L., Rennert G., Bonner J.D., Almog R., Tomsho L.P., Gruber S.B. (2004). BRCA1 and BRCA2 founder mutations and the risk of colorectal cancer. J. Natl. Cancer Inst..

[105] Van Asperen C.J., Brohet R.M., Meijers-Heijboer E.J., Hoogerbrugge N., Verhoef S., Vasen H.F., Ausems M.G., Menko F.H., Gomez Garcia E.B., Klijn J.G. (2005). Cancer risks in BRCA2 families: Estimates for sites other than breast and ovary. J. Med. Genet..

[106] Kadouri L., Hubert A., Rotenberg Y., Hamburger T., Sagi M., Nechushtan C., Abeliovich D., Peretz T. (2007). Cancer risks in carriers of the BRCA1/2 Ashkenazi founder mutations. J. Med. Genet..

[107] Phelan C.M., Iqbal J., Lynch H.T., Lubinski J., Gronwald J., Moller P., Ghadirian P., Foulkes W.D., Armel S., Eisen A. (2014). Incidence of colorectal cancer in BRCA1 and BRCA2 mutation carriers: Results from a follow-up study. Br. J. Cancer.

[108] Feliubadaló L., López-Fernández A., Pineda M., Díez O., Del Valle J., Gutiérrez-Enríquez S., Teulé A., González S., Stjepanovic N., Salinas M. (2019). Opportunistic testing of BRCA1, BRCA2 and mismatch repair genes improves the yield of phenotype driven hereditary cancer gene panels. Int. J. Cancer.

[109] Cullinane C.M., Creavin B., O’Connell E.P., Kelly L., O’Sullivan M.J., Corrigan M.A., Redmond H.P. (2020). Risk of colorectal cancer associated with BRCA1 and/or BRCA2 mutation carriers: Systematic review and meta-analysis. Br. J. Surg..

[110] Dobbins S.E., Broderick P., Chubb D., Kinnersley B., Sherborne A.L., Houlston R.S. (2016). Undefined familial colorectal cancer and the role of pleiotropism in cancer susceptibility genes. Fam. Cancer.

[111] Oh M., McBride A., Yun S., Bhattacharjee S., Slack M., Martin J.R., Jeter J., Abraham I. (2018). BRCA1 and BRCA2 Gene Mutations and Colorectal Cancer Risk: Systematic Review and Meta-analysis. J. Natl. Cancer Inst..

[112] Yurgelun M.B., Masciari S., Joshi V.A., Mercado R.C., Lindor N.M., Gallinger S., Hopper J.L., Jenkins M.A., Buchanan D.D., Newcomb P.A. (2015). Germline TP53 Mutations in Patients With Early-Onset Colorectal Cancer in the Colon Cancer Family Registry. JAMA Oncol..

[113] Hansen M.F., Johansen J., Sylvander A.E., Bjornevoll I., Talseth-Palmer B.A., Lavik L.A.S., Xavier A., Engebretsen L.F., Scott R.J., Drablos F. (2017). Use of multigene-panel identifies pathogenic variants in several CRC-predisposing genes in patients previously tested for Lynch Syndrome. Clin. Genet..

[114] Stoffel E.M., Koeppe E., Everett J., Ulintz P., Kiel M., Osborne J., Williams L., Hanson K., Gruber S.B., Rozek L.S. (2018). Germline Genetic Features of Young Individuals With Colorectal Cancer. Gastroenterology.

[115] Khan S.A., Idrees K., Forslund A., Zeng Z., Rosenberg S., Pincas H., Barany F., Offit K., Laquaglia M.P., Paty P.B. (2008). Genetic variants in germline TP53 and MDM2 SNP309 are not associated with early onset colorectal cancer. J. Surg. Oncol..

[116] Kratz C.P., Achatz M.I., Brugières L., Frebourg T., Garber J.E., Greer M.C., Hansford J.R., Janeway K.A., Kohlmann W.K., McGee R. (2017). Cancer Screening Recommendations for Individuals with Li-Fraumeni Syndrome. Clin. Cancer Res..

[117] Genetic/Familial High-Risk Assessment: Breast and Ovarian; Li-Fraumeni Syndrome. https://www.nccn.org/professionals/physician_gls/pdf/breast-screening.pdf.

[118] Wang L., Baudhuin L.M., Boardman L.A., Steenblock K.J., Petersen G.M., Halling K.C., French A.J., Johnson R.A., Burgart L.J., Rabe K. (2004). MYH mutations in patients with attenuated and classic polyposis and with young-onset colorectal cancer without polyps. Gastroenterology.

[119] Knopperts A.P., Nielsen M., Niessen R.C., Tops C.M., Jorritsma B., Varkevisser J., Wijnen J., Siezen C.L., Heine-Broring R.C., van Kranen H.J. (2013). Contribution of bi-allelic germline MUTYH mutations to early-onset and familial colorectal cancer and to low number of adenomatous polyps: Case-series and literature review. Fam. Cancer.

[120] Castillejo A., Vargas G., Castillejo M.I., Navarro M., Barbera V.M., Gonzalez S., Hernandez-Illan E., Brunet J., Ramon y Cajal T., Balmana J. (2014). Prevalence of germline MUTYH mutations among Lynch-like syndrome patients. Eur. J. Cancer.

[121] Segui N., Navarro M., Pineda M., Koeger N., Bellido F., Gonzalez S., Campos O., Iglesias S., Valdes-Mas R., Lopez-Doriga A. (2015). Exome sequencing identifies MUTYH mutations in a family with colorectal cancer and an atypical phenotype. Gut.

[122] Bellido F., Pineda M., Aiza G., Valdés-Mas R., Navarro M., Puente D.A., Pons T., González S., Iglesias S., Darder E. (2016). POLE and POLD1 mutations in 529 kindred with familial colorectal cancer and/or polyposis: Review of reported cases and recommendations for genetic testing and surveillance. Genet. Med..

[123] Ngeow J., Heald B., Rybicki L.A., Orloff M.S., Chen J.L., Liu X., Yerian L., Willis J., Lehtonen H.J., Lehtonen R. (2013). Prevalence of germline PTEN, BMPR1A, SMAD4, STK11, and ENG mutations in patients with moderate-load colorectal polyps. Gastroenterology.

[124] Nieminen T.T., Abdel-Rahman W.M., Ristimaki A., Lappalainen M., Lahermo P., Mecklin J.P., Jarvinen H.J., Peltomaki P. (2011). BMPR1A mutations in hereditary nonpolyposis colorectal cancer without mismatch repair deficiency. Gastroenterology.

[125] Fernandez-Rozadilla C., Brea-Fernández A., Bessa X., Alvarez-Urturi C., Abulí A., Clofent J., Payá A., Jover R., Xicola R., Llor X. (2013). BMPR1A mutations in early-onset colorectal cancer with mismatch repair proficiency. Clin. Genet..

[126] Valle L., de Voer R.M., Goldberg Y., Sjursen W., Försti A., Ruiz-Ponte C., Caldés T., Garré P., Olsen M.F., Nordling M. (2019). Update on genetic predisposition to colorectal cancer and polyposis. Mol. Asp. Med..

[127] Archambault A.N., Su Y.R., Jeon J., Thomas M., Lin Y., Conti D.V., Win A.K., Sakoda L.C., Lansdorp-Vogelaar I., Peterse E.F.P. (2020). Cumulative Burden of Colorectal Cancer-Associated Genetic Variants Is More Strongly Associated With Early-Onset vs Late-Onset Cancer. Gastroenterology.

